# Drug Repurposing for Kala-Azar

**DOI:** 10.3390/pharmaceutics17081021

**Published:** 2025-08-06

**Authors:** Biljana Arsić, Budimir S. Ilić, Andreas Maier, Michael Hartung, Jovana Janjić, Jelena Milićević, Jan Baumbach

**Affiliations:** 1 Department of Chemistry, Faculty of Sciences and Mathematics, University of Niš, Višegradska 33, 18000 Niš, Serbia; 2 Institute for Computational Systems Biology, University of Hamburg, Albert-Einstein-Ring 8-10, 22607 Hamburg, Germany; andreas.maier-1@uni-hamburg.de (A.M.); michael.hartung@uni-hamburg.de (M.H.); jan.baumbach@uni-hamburg.de (J.B.); 3 Department of Chemistry, Faculty of Medicine, University of Niš, Blvd. Dr Zorana Đinđića 81, 18000 Niš, Serbia; 4 Faculty of Biology, University of Belgrade, Studentski trg 16, 11000 Belgrade, Serbia; jovanajanjicc@gmail.com; 5 Laboratory for Bioinformatics and Computational Chemistry, Institute of Nuclear Sciences “Vinča”, University of Belgrade, Mike Petrovića Alasa 12-14, 11000 Belgrade, Serbia; jdjordjevic@vin.bg.ac.rs; 6 Computational Biomedicine Lab, Department of Mathematics and Computer Science, University of Southern Denmark, 5230 Odense, Denmark

**Keywords:** visceral leishmaniasis, drug repurposing, molecular docking, molecular dynamics simulation, Rab5a, PTR1, STAT3 pathway, entecavir, valganciclovir, nifuroxazide

## Abstract

**Objective:** Visceral leishmaniasis (VL), a Neglected Tropical Disease caused by *Leishmania donovani*, remains insufficiently addressed by current therapies due to high toxicity, poor efficacy, and immunosuppressive complications. This study aimed to identify and characterize repurposed drugs that simultaneously target parasite-encoded and host-associated mechanisms essential for VL pathogenesis. **Methods:** Two complementary in silico drug repurposing strategies were employed. The first method utilized electron–ion interaction potential (*EIIP*) screening followed by molecular docking and molecular dynamics (MD) simulations targeting two *L. donovani* proteins: Rab5a and pteridine reductase 1 (PTR1). The second approach employed network-based drug repurposing using the Drugst.One platform, prioritizing candidates via STAT3-associated gene networks. Predicted drug–target complexes were validated by 100 ns MD simulations, and pharmacokinetic parameters were assessed via ADMET profiling using QikProp v7.0 and SwissADME web server. **Results:** Entecavir and valganciclovir showed strong binding to Rab5a and PTR1, respectively, with Glide Scores of −9.36 and −9.10 kcal/mol, and corresponding MM-GBSA ΔG_bind values of −14.00 and −13.25 kcal/mol, confirming their stable interactions and repurposing potential. Network-based analysis identified nifuroxazide as the top candidate targeting the host JAK2/TYK2–STAT3 axis, with high stability confirmed in MD simulations. Nifuroxazide also displayed the most favorable ADMET profile, including oral bioavailability, membrane permeability, and absence of PAINS alerts. **Conclusions:** This study highlights the potential of guanine analogs such as entecavir and valganciclovir, and the nitrofuran derivative nifuroxazide, as promising multi-target drug repurposing candidates for VL. Their mechanisms support a dual strategy targeting both parasite biology and host immunoregulation, warranting further preclinical investigation.

## 1. Introduction

Visceral leishmaniasis (VL), better known by its Hindi name kala-azar (meaning black fever), belongs to the group of Neglected Tropical Diseases (NTDs) caused mainly by protozoa *Leishmania donovani* [1]. Among NTDs, it is associated with high mortality [2]. The vector for the disease is a female sandfly, for whom a blood meal is necessary to develop and lay eggs [3]. VL spreads in southern Europe, Africa, the Middle East, and Central and South America [4], connected to sand fly distribution areas with average temperatures above 15.6 °C for at least three months per year [5]. The symptoms reported by the WHO (2024) [1] include weight loss, irregular bouts of fever, enlargement of the spleen and liver, and anemia. Recent clinical evidence suggests that VL can cause impairment of the peripheral nervous system, ocular involvement, and central nervous system complications, including meningoencephalitis [6]. Neurological abnormalities were identified in almost 46% of VL patients, mainly the sensation of burning feet and foot drop (less frequently), difficulties in walking, cranial nerve deficits, and deafness [7].

Current treatments include (1) pentavalent antimonials (meglumine antimoniate and sodium stibogluconate), toxic and effective at the same time; (2) amphotericin B deoxycholate, with common nephrotoxicity and serious toxic effects (hypokalemia and myocarditis); (3) lipid formulations of amphotericin B with less toxic effects compared to amphotericin B deoxycholate; (4) paromomycin (hepatotoxicity can be developed); (5) pentamidine isethionate (severe adverse effects: shock, severe hypoglycemia, diabetes mellitus, myocarditis, and renal toxicity are limitations for its use); (6) miltefosine (induces gastrointestinal side-effects, potential teratogenic); (7) oral antifungal agents (ketoconazole, fluconazole, itraconazole) with variable effectiveness [1].

These seven options that are highly damaging to the patient’s system, including heavy side-effects combined with a rising number of incidents, leave an unmet medical need for effective but safer treatment options.

Drug repurposing is the effort to research new indications for already approved medicines or advance knowledge on previously studied but unapproved drugs. It drew attention during the COVID-19 pandemic after the FDA granted emergency use authorization (EUA) for some repurposed drugs to treat COVID-19 [8]. Repurposing drugs can be faster, cheaper, less risky, and more successful than usual drug development approaches because of the absence of the earlier stages of development that study drug safety, as they have already been performed [9]. Studies indicate that de novo drug discovery and development can take longer (10–17 years) than repurposed medicines, mostly approved within 3–12 years and at about half the cost [10].

So far, drug repurposing attempts for kala-azar has not been reported as frequently as for other diseases such as heart disease and cancer or other blockbuster diseases [11]. In one such attempt, in a previously published study, three commercially available kinase inhibitors (sunitinib, lapatinib, and sorafenib) showed IC_50_ values of 1, 2–3, and 3–4 µM, respectively, against *L. donovani* amastigotes in cultured murine macrophages, highly comparable to that of miltefosine, one of the current treatments for VL, which had an IC_50_ value of 1.0 µM [12]. Other drugs, such as sitamaquine, originally developed for malaria, have shown significant activity against *L. donovani* [13]. Some other reported attempts related to kala-azar included the repurposing of drugs, such as antifungals, antibiotics, anticancers, antidepressants, and antihypertensives [14], but with no successful outcomes yet.

Previously, we reported on several drug repurposing efforts, the most significant of which focused on COVID-19 [15] and antibiotic-related strategies [16]. Here, we aimed to identify drug repurposing candidates for kala-azar using two complementary computational approaches. The first method used consists of using the electron–ion interaction potential (*EIIP*) and molecular docking on two different protein targets from *L. donovani*: Rab5a and PTR1. The second method consists of using the drug repurposing platform Drugst.One (Figure S1), which integrates disease-associated gene networks for target prediction [17]. Drugst.One allows visualization, identification, and prioritization of drug targets and repurposing candidates based on a variety of network topology and centrality algorithms [17]. To validate the interactions found through docking and network-based screening, we utilized molecular dynamics (MD) simulations and molecular mechanics generalized Born surface area (MM-GBSA) binding free energy calculations to address conformational stability, flexibility, and temporal persistence of the protein–ligand complexes, under dynamic physiological conditions. Finally, we performed in silico ADMET profiling of all prioritized compounds to assess pharmacokinetic properties and drug-likeness [18]. These data were used to rationalize the translational potential of each compound, especially in the context of intracellular parasitic infections like visceral leishmaniasis, where both host- and parasite-targeting properties are essential.

## 2. Materials and Methods

### 2.1. Repurposing Method I (EIIP and Molecular Docking)

The *EIIP* was introduced in 1972 [19] and provides a numerical expression of the specific recognition between interacting molecules at a distance of >5 Å. It is defined by the following equation:

EIIP = 0.25(Z*/(2π))sin(1.04πZ*)

where Z* is the *AQVN* (Average Quasi Valence Number) determined by the following equation:

$$Z^{*}=\frac{1}{N}\sum_{i=1}^{m}n_{i}Z_{i}$$

where *Z_i_* is the valence number of the *i*-th atomic component, *n_i_* is the number of atoms of the *i*-th component, *m* is the number of atomic components in the molecule, and *N* is the total number of atoms. Z* and *EIIP* are expressed in Rydberg units (Ry), representing unique physical properties that, among molecular descriptors, characterize the long-range interactions between molecules [20]. Further, both have been shown to correlate significantly with the molecules’ biological activities (antibiotic activity, mutagenicity, carcinogenicity, etc.) [21].

*EIIP* calculations were performed on FDA-approved drugs retrieved from the ZINC database [22] and on guanosine triphosphate (GTP) and guanosine diphosphate (GDP), native ligands of Rab5a [23]. Compounds with *EIIP* values within ±20% of GTP and GDP were selected for molecular docking. Ligands were prepared with mainly the MacroModel module, and the LigPrep module only in molecules containing boron, in Schrödinger Suite 2022-3. The structure of the Rab5a protein (PDB ID: 6L6O) [23] was retrieved from the Protein Data Bank (PDB) and prepared using the Protein Preparation Wizard. Molecular docking was performed using Glide against the binding site of GTP in Rab5a [23].

A 3D structural model of pteridine reductase (PTR1) from *L. donovani*, based on its FASTA sequence [24], was generated in silico using the I-TASSER server [25]. The model with the highest C-score (0.36) was chosen for molecular docking to further filter candidates preselected based on *EIIP* values, using monastrol, a known pteridine reductase inhibitor, as a reference compound [26]. Docking was performed in the same manner as previously described for Rab5a. Candidate ligands were prepared using the MacroModel module of Schrödinger Suite 2022-3 and included energy minimization and conformational analysis. Only the global energy minimum conformation of each compound was used for docking.

### 2.2. Repurposing Method II Using the Online Platform Drugst.One

A list of kala-azar-associated genes was downloaded from the DisGeNET database [27]. Active human genes relevant to the disease were identified using the DOMINO algorithm [28,29], based on four different interactome (protein–protein) network datasets: DIP [30], HuRI [31], STRING [32], and PCNet [33]. Drug repurposing analysis was conducted *via* the Drugst.One platform using the Drug Search module, where four different algorithms (TrustRank, Harmonic Centrality, Degree Centrality, and Network Proximity) were applied twice, once for approved drugs only and once for a combination of approved and unapproved drug sets [17]. Interaction data were retrieved from multiple sources depending on interaction type: NeDRex (all types), STRING and IntAct (protein–protein), ChEMBL (drug–protein), DisGeNET (protein–disease), and DrugCentral (drug–disease). Indirect drug associations were also included. The resulting .graphml files were analyzed using Cytoscape 3.10.2 [34]. In addition, Drug Target Search was performed using kala-azar gene input and algorithms including multi-Steiner Trees [35], KeyPathwayMiner [36], and the previously mentioned centrality-based algorithms.

### 2.3. Molecular Dynamics Simulations and MM-GBSA Analysis of Protein–Ligand Complexes

Molecular dynamics (MD) simulations were performed to explore the conformational stability, flexibility, and dynamic nature of the critical protein–ligand complexes identified *via* docking studies and a network-based drug repurposing approach. All simulations were performed in the Desmond molecular dynamics program (version 2018.4, Schrödinger Inc., New York, NY, USA), developed by D.E. Shaw Research, under standard conditions. The protein structure and ligand conformations for each simulation were prepared via the same refinement and data preprocessing protocols established during molecular docking, including explicit assignment of protonation states, bond order correction, and removal of crystallographic artifacts.

The entecavir-Rab5a complex is based upon the crystal structure of *L. donovani* Rab5a (PDB ID: 6L6O) as published by Zohib et al. [23], while our valganciclovir–PTR1 complex utilized a homology model of *L. donovani* PTR1 from the I-TASSER server based upon the homologous FASTA sequence [24]. The nifuroxazide-JAK2 and nifuroxazide-TYK2 complexes utilized a high-resolution crystal structure of the JAK2 kinase domain (PDB ID: 7Q7I) and TYK2 (PDB ID: 8TB6), structures previously published by Wellaway et al. [37] and Breinlinger et al. [38], respectively.

Each of the protein–ligand complexes was placed into its own orthorhombic simulation box, solvated in explicit water using the simple point charge (SPC) model, and neutralized by adding appropriate counterions (Cl^−^ or Na^+^), as needed. The solvated systems, which consisted of ~20,000–40,000 atoms depending on the complex, were initially energy-minimized and followed by the standard six-step Desmond relaxation protocol to stabilize the system before production. Equilibrated systems were then subjected to 100 ns of MD simulations in an isothermal–isobaric (NPT) ensemble. The temperature was maintained at 300 K using a Nosé–Hoover thermostat, and the pressure was maintained at 1.01325 bar using a Martyna–Tobias–Klein barostat. Atomic coordinate data and system energies were saved every 10 ps during the simulations. Post-simulation analyses were performed using the simulation interaction diagram tools embedded in Maestro (Schrödinger Suite). The root mean square deviation (RMSD), used to assess overall temporal stability of the protein–ligand complexes, and the root mean square fluctuation (RMSF), which conceptualizes local residue-level flexibility, both indicated values consistently below 2 Å for each system, indicating that conformational dynamics was stable according to Liu and Kokubo [39]. Collectively, these data confirm the stability of the predicted binding modes under dynamic physiological conditions.

To further quantify binding affinities and validate docking predictions under physiologically relevant conditions, molecular mechanics generalized Born surface area (MM-GBSA) binding free energy calculations were performed using the thermalized MD trajectories. For the PTR1 and Rab5a targets, the top five ligands identified based on their Glide Scores were selected for post-docking evaluation. The Prime MM-GBSA module was employed to compute ΔG_bind for each ligand–protein complex. Snapshots were extracted every 1 ns from the 100 ns trajectories, and binding energies were averaged across these frames. This methodology integrates van der Waals, electrostatic, solvation, and ligand strain components to generate a refined estimation of the free energy of binding. More favorable (i.e., more negative) ΔG_bind values observed for select repurposed drugs—including entecavir, valganciclovir, and nifuroxazide—relative to the corresponding reference or endogenous ligands further support their potential as stable and effective inhibitors of their respective targets under dynamic conditions.

### 2.4. In Silico ADMET Profiling of Repurposed Compounds

In silico ADMET profiling was performed for entecavir, valganciclovir, and nifuroxazide using QikProp v7.0 (Schrödinger, LLC, New York, NY, USA). The following pharmacokinetic and physicochemical descriptors were computed: molecular weight (MW), number of rotatable bonds (RB), dipole moment (DM), total solvent-accessible volume (MV), estimated numbers of hydrogen-bond donors (DHB) and acceptors (AHB), van der Waals polar surface area (PSA), predicted octanol/water partition coefficient (logP), aqueous solubility (logS), Caco-2 cell permeability (PCaco), number of likely metabolic reactions (PM), and predicted human oral absorption (%HOA). Rule-based filters were applied to assess compliance with Lipinski’s rule of five (VRF) and Jorgensen’s rule of three (VRT). To assess assay interference risk, PAINS (pan-assay interference compounds) alerts were independently evaluated using the SwissADME web server [40]. All parameters were calculated using default settings and the results were used to support compound prioritization based on predicted bioavailability, metabolic stability, and overall drug-likeness in the context of VL.

## 3. Results and Discussion

### 3.1. Repurposing Method I

This study targeted two proteins of *L. donovani*, Rab5a and pteridine reductase 1 (PTR1), as potential drug repurposing targets due to their essential biological functions and prior use in visceral leishmaniasis [23,26]. Rab5a is a member of the Rab family of GTPase proteins involved in early endosomal trafficking essential for host-derived nutrient uptake and intracellular movement [23,41]. Functional studies have indicated that genetic ablation of Rab5a is lethal to the parasite, highlighting its essential role for parasite survival and supporting its potential as a druggable target in kala-azar [42]. PTR1 is an essential enzyme in the folate biosynthetic pathway of *Leishmania* species as it serves as a metabolic bypass when dihydrofolate reductase is inhibited [26]. Previously, monastrol, originally developed as an anticancer agent [43], demonstrated potent antileishmanial activity in vitro and in vivo using a hamster model of visceral leishmaniasis [26].

**Figure 1:**
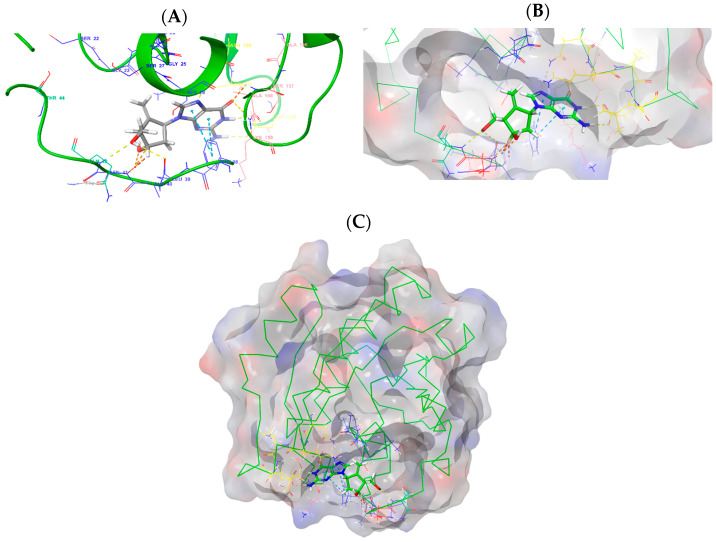
(**A**) Docking conformation of entecavir within the Rab5a binding site of *L. donovani*; (**B**) the surface view of the binding pocket showing bound entecavir; (**C**) the complete surface model of Rab5a with entecavir positioned in the active site.

The initial *EIIP*-based screening of approved drugs targeting Rab5a, using GTP and GDP as reference compounds, was performed using the ZINC database [22]. This yielded candidate compounds, which were subsequently filtered based on their Glide Scores [44,45,46] obtained through molecular docking. Glide was chosen because it is a high-accuracy algorithm with systematic sampling of the ligand’s orientation, conformation, and position in the binding site that outperformed traditional docking algorithms [44,45,46]. With a Glide Score of −9.36 kcal/mol, entecavir demonstrated a stronger predicted interaction with Rab5a than GTP (Figure 1). This finding was substantiated by MM-GBSA binding free energy results (ΔG_bind = −14.00 kcal/mol for entecavir vs. −13.04 kcal/mol for GTP), confirming its potential as a robust inhibitor (Table 1). Entecavir is currently approved for clinical use in the treatment of hepatitis B virus infection [47].

**Figure 2:**
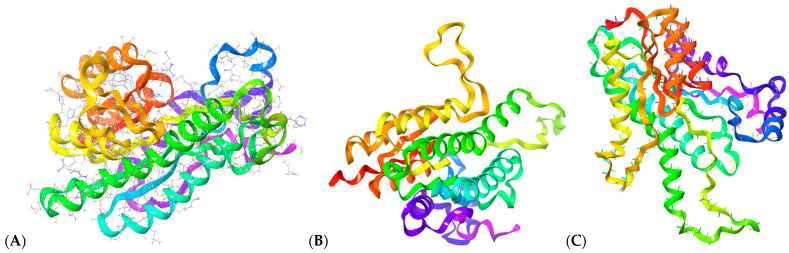
Structural models of *L. donovani* PTR1 generated using (**A**) I-TASSER, (**B**) AlphaFold, and (**C**) Rosetta.

Computational modeling of PTR1 as a drug target was more challenging than for Rab5a. The X-ray crystal structure of *L. donovani* PTR1 (PDB ID: 2XOX) was not usable for molecular docking, due to structural limitations [48], which required us to generate a working protein model using the I-TASSER server [25]; the c-score of this model was 0.36 (Figure 2A) [49]. The Z-score of the I-TASSER model calculated using ProSA-web [50] was −7.84 and less favorable than the original 2XOX structure (Z-score −6.41). The AlphaFold-predicted structure (Figure 2B) [51] showed a slightly lower Z-score of −8.21, while the Rosetta-generated model (Figure 2C) [52] exhibited the lowest Z-score at −8.46. While the AlphaFold and Rosetta structures had lower Z-scores than the I-TASSER structure did, the I-TASSER output was favored for dockings as it provided a more reliable active site prediction and was compatible with downstream molecular docking workflows [25].

Compounds from the ZINC database [22] that passed *EIIP*-based filtering, using monastrol as the reference ligand [26], were further evaluated by molecular docking. Valganciclovir, a clinically approved antiviral [53], exhibited the highest Glide Score against PTR1 (−9.097 kcal/mol), which was consistent with its MM-GBSA binding free energy of −13.25 kcal/mol, supporting its prioritization as a strong repurposing candidate (Table 2).

### 3.2. Repurposing Method II

Given that the mechanisms of action for many current antileishmanial therapies remain insufficiently understood [54], we opted for a complementary strategy based on network pharmacology and disease-associated gene profiling using the Drugst.One platform.

In this drug repurposing study, active genes were entered as nodes identified by DOMINO [28,29]. Notably, DIP [30] and HuRI [31] as interactome networks resulted in fewer active genes and candidate drugs for kala-azar. A higher number of active genes and drug candidates were predicted with STRING [32] and PCNet [33]. The first ranked candidates at the top 100 obtained using different databases and algorithms separately (TrustRank, Harmonic Centrality, Degree Centrality, and Network Proximity) are displayed in Figure S2. The top-ranked compounds spanned various therapeutic classes, including corticosteroids, oncology agents, antibiotics, cardiac glycosides, antihypertensives, anticoagulants, and treatments for anemia, leukemia, and asthma (Figure S2).

Further analysis of the top-ranked candidates (Figure S2) revealed functional associations with several disease-relevant genes, including STAT3, IL5, ITGB2, HBA1, INPP5D, MMP9, and LCK, as shown in Figure S3.

Across multiple network-based algorithms (Network Proximity, Degree Centrality, TrustRank, and Harmonic Centrality), STAT3 scored highest as a drug target for both STRING and PCNet interaction networks. After removing STAT3 from the input gene list, mostly only few candidate drugs were recovered with the algorithms of Network Proximity and Degree Centrality, and only partial recovery occurred with Harmonic Centrality and TrustRank. The Drugst.One platform highlighted STAT3 as a central regulatory node in visceral leishmaniasis pathogenesis (Table 3), and suggested that it might coordinate important immunosuppressive pathways that *L. donovani* takes advantage of. This is strongly evidenced by Biswas et al.’s study [55] that provides empirical evidence that activation of STAT3 by IL-10-mediated pathways enhances IL-4Rα expression, which induces arginase 1 and indirectly suppresses macrophage clearance of parasites. With our observations of STAT3 as a meaningful driver of the network, aligned with the empirical data, we suggest that targeted manipulation of STAT3 could relieve the immunosuppressive response and decrease *Leishmania* survival in host macrophages.

Nifuroxazide was the most frequently prioritized candidate in our analysis, is currently approved for acute diarrhea and traveller’s colitis [56], and has also been reported to have encouraging activity against *L. donovani* [57]. Table 3 shows that nifuroxazide was identified in 22 of the 23 network-based scenarios (95.7%) and was clearly distinguished from every other drug considered. Nifuroxazide has been shown to inhibit the STAT3 signaling pathway *via* the inhibition of upstream kinases JAK2 and TYK2, and thus prevent the tyrosine phosphorylation of STAT3 and ultimately its transcriptional activity [58]. This was confirmed in multiple tumor models where nifuroxazide inhibited upstream JAK2 and TYK2, thus decreasing the phosphorylation of STAT3 and inhibiting tumor growth and metastases. Examples include models of breast cancer [59], melanoma [60], and colorectal carcinoma [61], supporting the therapeutic potential of nifuroxazide attributable to inhibition of STAT3. These findings are especially noteworthy given reports of the role of IL-10-mediated STAT3 activation in visceral leishmaniasis, which were reviewed by Samant et al. [62], who noted that dysregulation of the JAK/STAT pathway contributes to parasite persistence and immunosuppression. Because nifuroxazide was the top candidate in our network-based drug repurposing analysis, and given that we know that nifuroxazide can inhibit the JAK2/TYK2-STAT3 signal transduction axis, we might suggest that nifuroxazide might restore immune responsiveness in VL by interrupting this pathogenic signaling cascade.

Network-based drug repurposing analysis also considered the antiparasitic agents pyrimethamine and niclosamide as the second-best candidates potentially working on the STAT3 pathway, with prioritization scores of only 34.8% compared to 95.7% for nifuroxazide (Table 3) indicating the potential advantage of nifuroxazide. Furthermore, experimental evidence supports that pyrimethamine and niclosamide inhibit STAT3 signaling by different mechanisms. Pyrimethamine interrupts STAT3-dependent transcription by the inhibition of dihydrofolate reductase [63]. Niclosamide inhibits phosphorylation, as well as the nuclear translocation of STAT3 upstream of any known kinases, including JAK2, illustrating a mechanism for STAT3 inhibition independent of JAK2 [64].

Overall, the experimental studies noted above provide strong evidence to support the validity of our network-based drug repurposing method and, given the clear advantage of nifuroxazide in our analysis, we aim to investigate the interaction of nifuroxazide with experimentally confirmed STAT3-related targets, specifically upstream kinases JAK2 and TYK2, as a putative therapeutic strategy to combat visceral leishmaniasis, where dysregulation of the JAK/STAT pathway fuels parasite persistence and host immune suppression.

### 3.3. Binding Stability and Mechanistic Insights from Molecular Dynamics Simulations of Repurposed Guanine Analogs Targeting Rab5a and PTR1

**Figure 3:**
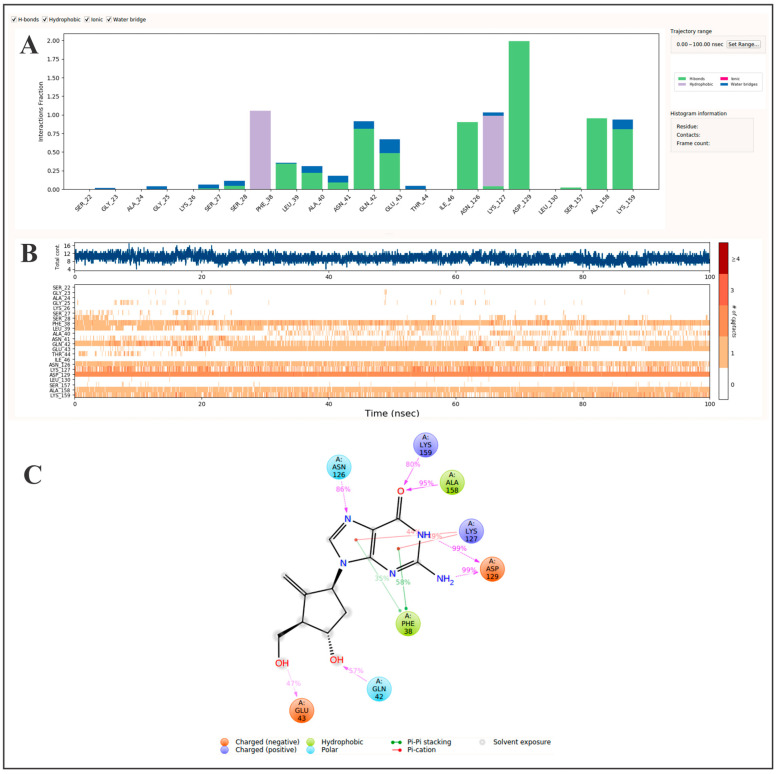
Molecular dynamics analysis of entecavir binding to Rab5a over 100 ns. (**A**) A protein–ligand interaction profile showing the frequency of key contact types: hydrogen bonds, hydrophobic interactions, ionic interactions, and water bridges. Interaction persistence is normalized across the trajectory, where a value of 1.0 indicates that contact was maintained for 100% of the simulation time. (**B**) An interaction timeline indicating when each amino acid residue interacts with the ligand during the simulation. The top panel displays the total number of simultaneous contacts at each time point, while the bottom panel maps specific residue interactions, with darker shading denoting stronger or multiple interactions. (**C**) A spatial contact map summarizing persistent ligand–residue interactions. Only interactions occurring in >30% of the simulation time are displayed. A single residue may contribute multiple interactions of the same type, which can yield values >100%. Collectively, these data offer mechanistic insights into the binding stability and interaction hotspots relevant for Rab5a inhibition.

Here, we investigated the repurposing potential of entecavir, a clinically approved antiviral agent and guanine analog, as a novel Rab5a inhibitor for targeting *L. donovani*. Molecular docking and molecular dynamics simulations indicated that entecavir has a greater binding affinity to Rab5a than GTP and maintained a stable binding interaction in a 100 ns simulation (Table 1, Figure 3). In support of this observation, fractional interaction analysis (Figure 3A) indicated significant and maintained hydrogen and hydrophobic bonding interactions with the residues Phe38 (1.05), Gln42 (0.92), Glu43 (0.68), Asn126 (0.90), Lys127 (1.03), Asp129 (1.99), Ala158 (0.95), and Lys159 (0.93). These residues are located near the switch I and II regions of Rab5a, which are essential to GDP/GTP exchange, effector protein interaction, and conformational cycling [65]. The guanosine-like scaffold of entecavir is responsible for its binding affinity toward Rab5a by replicating certain essential features of the native GTP ligand and engages with residues Phe38, Asn126, Lys127, Asp129, Ala158, and Lys159, shown in Figure 3C. Given that *L. donovani* Rab5a was shown to be a key regulator of vesicular trafficking and early endosome organization in the parasite, it is reasonable to predict that a pharmacological blockade of GTP-binding functionality may disrupt intracellular survival capabilities. As previously stated, *L. donovani* increases Rab5a expression, thereby re-shaping early endosomes to escape later phagolysosomal degradation [66]. Importantly, comparative molecular dynamics simulations revealed that entecavir exhibits more stable hydrogen-bonding and π-stacking interactions than GTP, with reduced conformational flexibility and sustained engagement of key pharmacophoric residues. These dynamic features were further corroborated by MM-GBSA binding free energy calculations, which indicated a more favorable ΔG_bind for entecavir relative to GTP (Table 1). Collectively, these findings reinforce entecavir’s potential to stabilize Rab5a in an inactive conformation, thereby interfering with critical endosomal processes and offering a promising strategy for therapeutic intervention in visceral leishmaniasis.

Although direct studies investigating Rab5a inhibition are limited, there are structure-based studies that have indicated useful information regarding Rab5a binding pockets, and pharmacophoric properties. Edler and Stein [65] conducted a molecular dynamics study confirming that a membrane-bound Rab5 is a druggable target, especially in scenarios where small molecules stabilize the inactive or transitional states of Rab5 and disrupt its membrane dynamics. Zhang et al. [67] identified neoandrographolide as an inhibitor interacting with Rab5 through amino acid residues distinct from those highlighted in our study, but still proximal to the switch I and II regions, disrupting endosomal fusion and trafficking pathways crucial for cancer progression. Similarly, Takeda et al. [68] demonstrated that the anti-malarial drug mefloquine hydrochloride effectively inhibits Rab5 in cancer treatment, reinforcing the rationale that entecavir, an antiviral agent, could analogously be repurposed for targeting Rab5a in leishmaniasis.

We further explored drug repurposing by evaluating valganciclovir, a guanine analog, as a potential direct inhibitor of *Leishmania* PTR1, a key enzyme in parasite folate metabolism and drug resistance [69,70]. While entecavir targeted the *Leishmania*-derived Rab5a GTPase, implicated in parasite vesicular trafficking, valganciclovir was investigated for its ability to bind directly to the parasite enzyme PTR1 and interfere with its catalytic function.

**Figure 4:**
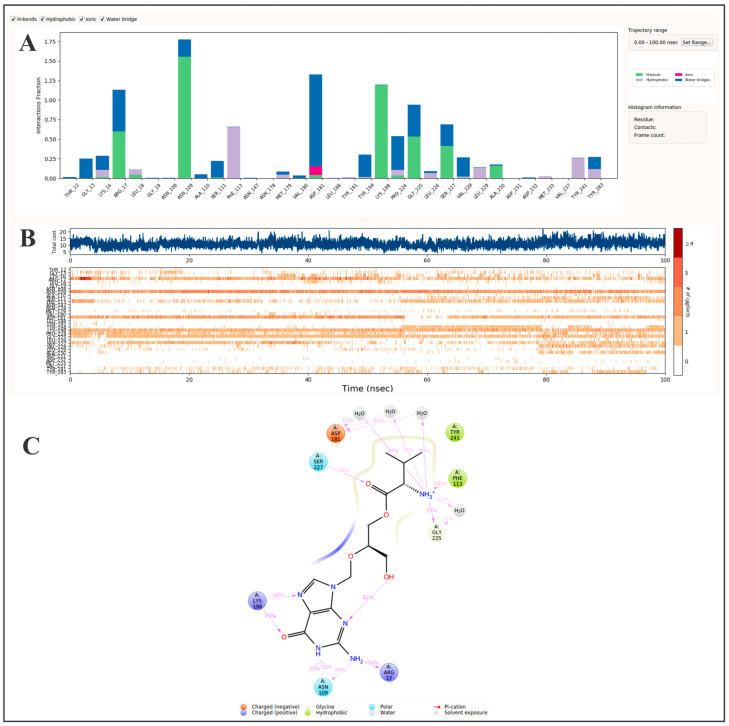
Molecular dynamics analysis of valganciclovir binding to PTR1 over 100 ns. (**A**) The protein–ligand interaction profile showing the frequency of key contact types: hydrogen bonds, hydrophobic interactions, ionic interactions, and water bridges. Interaction persistence is normalized across the trajectory, where a value of 1.0 indicates that a contact was maintained for 100% of the simulation time. (**B**) An interaction timeline indicating when each amino acid residue interacts with the ligand during the simulation. The top panel displays the total number of simultaneous contacts at each time point, while the bottom panel maps specific residue interactions, with darker shading denoting stronger or multiple interactions. (**C**) A spatial contact map summarizing persistent ligand–residue interactions. Only interactions occurring in >20% of the simulation time are displayed. A single residue may contribute multiple interactions of the same type, which can yield values >100%. Collectively, these data offer mechanistic insights into the binding stability and interaction hotspots relevant for PTR1 inhibition.

Molecular dynamics investigations of the PTR1–valganciclovir complex showed a stable binding pose and preserved interactions with residues of catalytic importance over a 100 ns trajectory (Table 2, Figure 4). Fractional interaction analysis (Figure 4A) showed that valganciclovir had high occupancy with Arg17 (1.13), Asn109 (1.78), Phe113 (0.65), Asp181 (1.33), Lys198 (1.19), Gly225 (0.95), and Ser227 (0.68). Additionally, the guanine-like moiety of valganciclovir appears to have established favorable hydrogen bonding and water-bridged interactions with Arg17, Asn109, and Lys198 (Figure 4C), which are characterized to stabilize NADP(H) and were described as part of the proton relay system in PTR1-catalyzed reactions [69,71]. What was revealed by the guanine-like moiety of valganciclovir in the molecular dynamics study resembled the previous interactions described in the structure by Dello Iacono et al. [69], with Arg17 and Asn109 as the principal residues targeting the NADP(H) position and Lys198 supporting the active-site Tyr194. In contrast, the opposite end of the molecule, bearing carbonyl and amine groups, engaged Phe113, Asp181, Gly225, and Ser227 through additional hydrogen bonds and water-bridged and hydrophobic interactions (Figure 4C). Overall, the site-specific engagements suggest that valganciclovir may simultaneously occupy both the cofactor- and substrate-binding regions of PTR1, potentially blocking its catalytic cycle through cooperative inhibition [69].

The identification of these residues aligns with what has been reported by Arthur et al. [71], who revealed that Arg17, Asn109, Phe113, Asp181, Lys198, Gly225, and Ser227 defined interaction hotspots required for PTR1 inhibition in their docking studies with natural product derivatives. A similar engagement of Arg17, Phe113, Asp181, and Lys198 was observed in chroman-4-one derivative complexes described by Di Pisa et al. [72]. Furthermore, our results support earlier mechanistic insights by Gourley et al. [70], who linked PTR1 inhibition to disruption of the folate cycle and increased drug susceptibility in trypanosomatid parasites. Overall, these data support our hypothesis that valganciclovir, through its guanine-like moiety, acts as a direct PTR1 inhibitor by mimicking NADP(H) and forming strong interactions with catalytic residues.

In contrast to entecavir, which obstructs endosomal dynamics mediated by *Leishmania* Rab5a, valganciclovir presents a more direct enzymatic blockade by binding to PTR1, which may lead to catalytic inactivation and disruption of the parasite’s folate metabolism [70]. Previous studies have further added to the antileishmanial potential of guanine-based scaffolds. Both valganciclovir and entecavir have a guanine-like moiety, and guanine and guanosine analogs have been found to inhibit *Leishmania* spp. effectively, as reported by Avila et al. [73], emphasizing the pharmacological validity of this chemotype. Similarly, formycin B, another guanine analog, exhibited proven efficacy by disrupting purine metabolism in *Leishmania*, as reported by Rainey and Santi [74]. These findings align with our computational observations and suggest that guanine-derived compounds like valganciclovir and entecavir could serve as effective inhibitors, either alone or as part of synergistic combinations. Indeed, the benefit of combining mechanistically distinct agents is highlighted by Intakhan et al. [75] and reviewed comprehensively by van Griensven et al. [76], emphasizing combination therapy as a rational direction for improving visceral leishmaniasis treatment outcomes.

### 3.4. Molecular Dynamics Characterization of Nifuroxazide as a JAK2/TYK2 Inhibitor for STAT3 Pathway Disruption in Visceral Leishmaniasis

STAT3 is a key transcriptional regulator involved in immune evasion during visceral leishmaniasis, where IL-10-induced signaling triggers its activation, leading to suppression of host inflammatory responses and promotion of parasite survival [55]. Targeting this induced immunosuppressive pathway has emerged as a feasible semi-therapeutic strategy, especially given the most recent research that has demonstrated the ability of cytokine-mediated regulation to restore effective immune responses in experimental and clinical models of visceral leishmaniasis [62].

**Figure 5:**
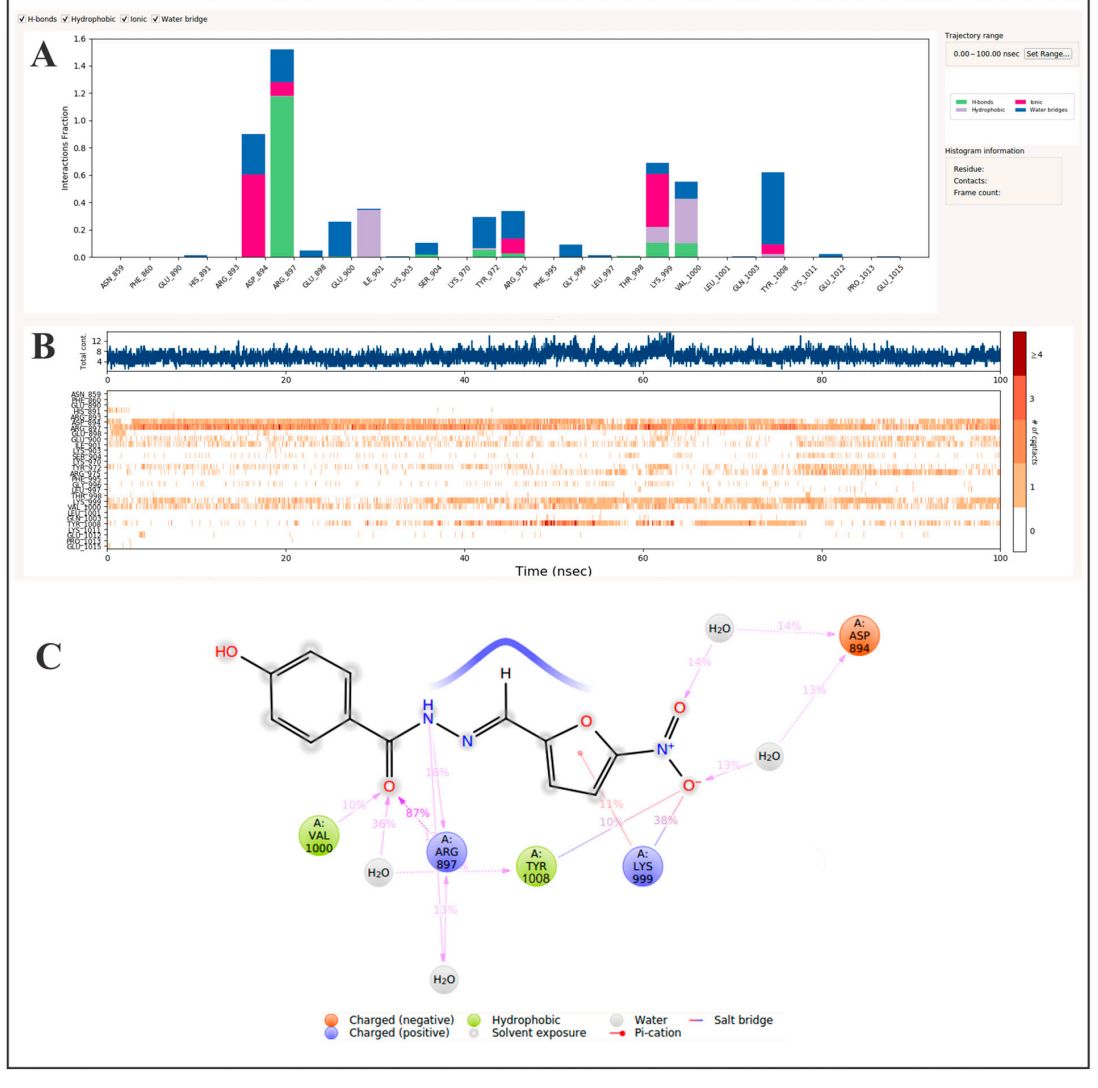
Molecular dynamics analysis of nifuroxazide binding to JAK2 over 100 ns. (**A**) A protein–ligand interaction profile showing the frequency of key contact types: hydrogen bonds, hydrophobic interactions, ionic interactions, and water bridges. Interaction persistence is normalized across the trajectory, where a value of 1.0 indicates that a contact was maintained for 100% of the simulation time. (**B**) An interaction timeline indicating when each amino acid residue interacts with the ligand during the simulation. The top panel displays the total number of simultaneous contacts at each time point, while the bottom panel maps specific residue interactions, with darker shading denoting stronger or multiple interactions. (**C**) A spatial contact map summarizing persistent ligand–residue interactions. Only interactions occurring in >10% of the simulation time are displayed. A single residue may contribute multiple interactions of the same type, which can yield values >100%. Collectively, these data offer mechanistic insights into the binding stability and interaction hotspots relevant for JAK2 inhibition.

**Figure 6:**
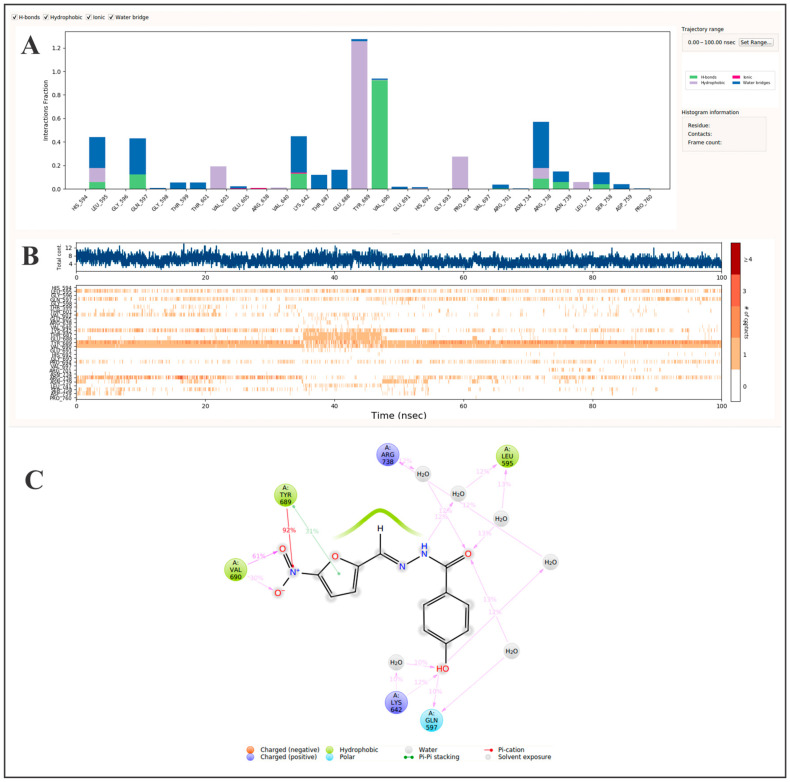
Molecular dynamics analysis of nifuroxazide binding to TYK2 over 100 ns. (**A**) A protein–ligand interaction profile showing the frequency of key contact types: hydrogen bonds, hydrophobic interactions, ionic interactions, and water bridges. Interaction persistence is normalized across the trajectory, where a value of 1.0 indicates that a contact was maintained for 100% of the simulation time. (**B**) Interaction timeline indicating when each amino acid residue interacts with the ligand during the simulation. The top panel displays the total number of simultaneous contacts at each time point, while the bottom panel maps specific residue interactions, with darker shading denoting stronger or multiple interactions. (**C**) Spatial contact map summarizing persistent ligand–residue interactions. Only interactions occurring in >10% of the simulation time are displayed. A single residue may contribute multiple interactions of the same type, which can yield values >100%. Collectively, these data offer mechanistic insights into the binding stability and interaction hotspots relevant for TYK2 inhibition.

Nifuroxazide, a nitrofuran-based antidiarrheal agent, has been identified as a potent STAT3 inhibitor in various tumor models, where it impairs STAT3 phosphorylation by inhibiting upstream JAK2 and TYK2 activity [58,59,60,61]. The molecular basis for this inhibition, however, has remained underexplored in infectious disease contexts. Given that structurally related nitrofurans such as nifurtimox exhibit established antiparasitic efficacy against *Leishmania* spp. and *Trypanosoma cruzi* [77,78], and that nifuroxazide was prioritized in our computational drug repurposing pipelines for leishmaniasis (Table 3), we performed MD simulations to investigate its interaction with the JAK2 and TYK2 kinases (Figure 5 and Figure 6).

Molecular dynamics simulations of the nifuroxazide–JAK2 complex showed persistent interaction of nifuroxazide with stable occupancy of the binding pocket, which is located within the ATP-binding cleft of the JH1 catalytic domain [79]. During the molecular dynamics simulation, a number of significant interactions were observed with residues Asp894 (0.90), Arg897 (1.52), Lys999 (0.69), Val1000 (0.56), and Tyr1008 (0.62) (Figure 5A). These interactions included hydrogen bonds, salt bridges, hydrophobic contacts, and water-mediated bridges which demonstrate a stable and specific binding mode. Most importantly, the nitrofuran moiety interacted with Asp894, Lys999, and Tyr1008, while the amide tail region engaged with Arg897 and Val1000 (Figure 5C), which indicates a bifunctional anchoring pattern that stabilizes the ligand in the JH1 active site. The relevance of these residues is highlighted by multiple independent studies. In the recent MD modeling study, using a milestoning-based Markovian methodology, Asp894 and Arg897 were highlighted as key interaction residues for JAK2 inhibitors [79]. Furthermore, Lys999, Val1000, and Tyr1008 were identified as contributing to the inhibitor specificity in co-crystal structures of JAK2 with other ATP-competitive inhibitors [80]. These studies validate our simulation results, and place nifuroxazide within the pharmacophoric envelope that has been defined by other JH1-directed agents.

From a medicinal chemistry standpoint, the insights presented here add complementary knowledge to the previously understood bioactivity of nitrofurans. The nitroaromatic core of nifuroxazide undergoes reductive activation in pathogens, producing reactive intermediates that generate oxidative stress [77,81], while derivatization strategies such as those employed for nitrofuranylazines have yielded leads with potent anti-*Leishmania* activity [78]. Importantly, the dual action of nifuroxazide, direct inhibition of JAK2 and indirect STAT3 pathway modulation, may explain its superior network prioritization as a repurposed candidate for leishmaniasis (Table 3).

After obtaining the interaction profile of nifuroxazide with JAK2, and with previous studies confirming that nifuroxazide inhibition of STAT3 phosphorylation is mediated through inhibition of JAK2 and TYK2 [58], we subsequently tested the binding characteristics for nifuroxazide within the TYK2 domain. The molecular dynamics simulation showed that the compound maintained interactions within the ATP-binding site, where the most significant interactions were exhibited with Tyr689 (1.28) and Val690 (0.93), which were mediated by the nitrofuran moiety via hydrophobic and hydrogen bond interactions, respectively (Figure 6A). The analysis revealed further stabilizing contacts with Leu595 (0.44), Gln597 (0.43), Lys642 (0.45), and Arg738 (0.57), which were mediated primarily through water bridge contacts. In particular, Leu595 interacted with the terminal amide group of nifuroxazide, while Gln597, Lys642, and Arg738 formed transient contacts with the phenolic group (Figure 6C). The observed interaction profile reflects binding behaviors characteristic of structurally validated TYK2 inhibitors [82,83,84,85], and supports the proposed role of nifuroxazide in modulating STAT3 activation *via* TYK2 engagement.

These mechanistic findings support a novel paradigm for nitrofuran-based immunochemotherapy: targeting host immunoregulatory pathways hijacked by *Leishmania* spp., in addition to classical parasite-directed oxidative mechanisms. Given the importance of IL-10/STAT3 signaling in parasite persistence [55], disruption of the JAK2/TYK2-STAT3 interface by nifuroxazide may not only impair parasite immune evasion but also synergize with antiparasitic stress induced by its redox-active core.

### 3.5. Translational Relevance of ADMET Properties in Repurposed Drug Candidates Identified Through Molecular Dynamics and Network-Based Prioritization

To complement the molecular docking and dynamics results presented above, we performed a more complete in silico ADMET assessment of entecavir, valganciclovir, and nifuroxazide (Table 4). These parameters provide essential context for assessing each drug’s translational potential, especially in the setting of intracellular parasitic infections such as visceral leishmaniasis, where both host- and parasite-directed mechanisms are relevant.

Of the drugs under investigation, nifuroxazide has the most pharmacokinetically advantageous profile. Based on high predicted intestinal permeability (PCaco = 62.9 nm/s), reasonably balanced lipophilicity (logP = 1.3), and predicted human oral absorption of 67%, there is strong evidence to expect effective oral bioavailability (Table 4). Critically, it adheres to both Lipinski’s rule of five and Jorgensen’s rule of three, indicating optimal physicochemical and biopharmaceutical properties. These features are especially relevant in the context of its dual-target action on host JAK2 and TYK2 kinases, where intracellular accumulation is required for STAT3 pathway modulation [86]. Finally, nifuroxazide had a low number of predicted metabolic reactions (PM = 2) indicating potential metabolic stability and perhaps a longer systemic half-life. Nonetheless, it is important to emphasize that the current clinical use of nifuroxazide is limited to the gastrointestinal tract, where it functions as a locally acting intestinal antiseptic. As reported in several studies compiled within reference [81], nifuroxazide exhibits poor gastrointestinal absorption and minimal systemic exposure in conventional formulations and dosages. This contrast between in silico predictions and real-world pharmacokinetics highlights the importance of future formulation efforts and experimental validation to assess its potential for repurposing in systemic and intracellular applications.

On the other hand, despite showing a strong and lasting interaction with *Leishmania* PTR1, valganciclovir has multiple ADMET-related liabilities. With a very high polar surface area (182.2 Å^2^), a large number of hydrogen bond donors and acceptors, and poor lipophilicity (logP = −1.6), it suffers from poor permeability (PCaco = 3.8 nm/s) and a very low predicted oral absorption of just 2% (Table 4). It violates both Lipinski and Jorgensen rules which indicate poor passive absorption with low bioavailability. These results suggest that, although valganciclovir’s mode of binding within the PTR1 active-site was structurally therapeutically sound, its physicochemical limitations may require some formulation efforts through prodrug derivatization or encapsulation in lipid-based delivery systems, to ensure a therapeutic effect [87]. It is also important to note that valganciclovir requires hepatic activation to convert into its active metabolite, ganciclovir. Since the liver is the primary organ targeted by *Leishmania* parasites during visceral infection, this dual role as both the metabolic site of drug activation and the parasite reservoir presents a unique translational challenge. The pharmacokinetic behavior of valganciclovir in infected hepatic tissue remains unknown and warrants future experimental investigation to assess whether its activation and intracellular accumulation are sufficiently robust under pathophysiological conditions.

Entecavir occupies an intermediate position in terms of ADMET performance. It has a good molecular weight and solubility (logS = −2.3), poor permeability (PCaco = 22.6 nm/s), and a moderate oral bioavailability (45%), which could limit systemic availability (Table 4). However, given its strong interaction with *L. donovani* Rab5a switch regions and the parasite-directed mechanism, even partial systemic bioavailability could be sufficient to disrupt endosomal trafficking and compromise intracellular parasitic survival. Further medicinal chemistry modification or formulation could enhance its pharmacokinetic profile [88].

Importantly, none of the compounds generated PAINS alerts (Table 4), reducing the chance of false-positive results in phenotypic assays, while also implying their target engagement specificity [89]. In addition to the ADMET advantages, nifuroxazide’s bifunctional inhibition of JAK2 and TYK2 supports its potential therapeutic value in repurposing as an immunomodulatory agent for treating leishmaniasis. Although in silico ADMET models provide excellent initial phase information, they are ultimately only algorithmic estimates and lack experimental validation, requiring additional in vitro and in vivo studies for a reliable translation [90]. In addition, we will need further pharmacodynamic studies, including dose–response characterization and cytotoxicity on infected macrophage cell lines, to develop therapeutic windows and selectivity indices.

## 4. Conclusions

Our research highlights the therapeutic potential for targeting both parasite-specific and host-directed pathways in visceral leishmaniasis. Entecavir and valganciclovir target critical parasite-derived intracellular processes, specifically Rab5a-mediated vesicular trafficking and PTR1-based folate metabolism, while nifuroxazide targets the host-derived JAK2/TYK2–STAT3 immune suppressive signaling pathway used by *Leishmania* to survive. This dual targeting approach provides a strong basis for drug repurposing, while also incorporating antivirals, anticancer, and antiparasitic pharmacology. While our in silico findings support the structural and biopharmaceutical potential of several repurposed drugs for antileishmanial therapy, it is essential to underscore that these predictions require experimental validation. The discrepancy between computational affinity estimates and in vitro/in vivo efficacy is a well-documented challenge, and many compounds with promising profiles may ultimately fail due to poor cellular uptake, off-target effects, or lack of metabolic stability. Future work will focus on validating these candidates in cell-based and enzymatic assays, followed by optimization efforts guided by the presented MD and MM-GBSA insights. Only through such integrative strategies can the attrition rate in antileishmanial drug development be reduced and new therapeutic leads effectively translated toward clinical relevance.

## Figures and Tables

**Table 1:** *EIIP* values, Glide Scores, and MM-GBSA binding free energies of top-ranked compounds and the reference ligand targeting Rab5a, along with their therapeutic uses.

Drugs	*EIIP* (Ry)	Glide Score (kcal/mol)	ΔG_bind (kcal/mol)	Use
Entecavir	−0.0546	−9.360	−14.00	Antiviral
Acyclovir	−0.1054	−8.513	−9.32	Antiviral
Gancyclovir	−0.0988	−8.132	−10.42	Antiviral
Eltrombopag	−0.0508	−7.830	−12.48	TPO receptor agonist
Zileuton	−0.0439	−7.808	−10.90	Anti-inflammatory
GTP	−0.0461	−9.094	−13.04	Reference ligand

**Table 2:** *EIIP* values, Glide Scores, and MM-GBSA binding free energies of top-ranked compounds targeting PTR1, along with their therapeutic uses.

Drugs	*EIIP* (Ry)	Glide Score (kcal/mol)	ΔG_bind (kcal/mol)	Use
Valganciclovir	−0.0196	−9.097	−13.25	Antiviral
*(S)*-Rosiglitazone	−0.0266	−8.327	−11.14	Antidiabetic
Fosclevudine alafenamide	−0.0264	−7.574	−12.85	Antiviral
Apixaban	−0.0246	−7.146	−10.00	Anticoagulant
Zebinix	−0.0239	−6.986	−11.56	Antiepileptic

**Table 3:** The top 100 drug candidates associated with the STAT3 gene identified using the Drugst.One platform.

Interactome Used	Algorithm Used	Databases Used	Drugs (to Be Considered)-STAT3
STRING	Network Proximity	NeDRex	nifuroxazide
STRING	Network Proximity	STRING (P-P), NeDRex (D-P, D-D), DisGeNET (P-D)	nifuroxazide
STRING	Harmonic Centrality	NeDRex	ouabain, digoxin, niclosamide, digitoxin, nifuroxazide
STRING	TrustRank	NeDRex	ouabain, digoxin, niclosamide, digitoxin, nifuroxazide
STRING	TrustRank	IntAct (P-P), ChEMBL (D-P), DisGeNET (P-D), DrugCentral (D-D)	diosmin, acetylcysteine, nifuroxazide, pyrimethamine
STRING	Harmonic Centrality	IntAct (P-P), ChEMBL (D-P), DisGeNET (P-D), DrugCentral (D-D)	acetylcysteine, diosmin, nifuroxazide, pyrimethamine
STRING	Degree Centrality	IntAct (P-P), ChEMBL (D-P), DisGeNET (P-D), DrugCentral (D-D)	nifuroxazide, pyrimethamine, acetylcysteine, diosmin
STRING	Network Proximity	IntAct (P-P), ChEMBL (D-P), DisGeNET (P-D), DrugCentral (D-D)	ciglitazone, genistein, pyrimethamine, acetylcysteine, diosmin, nifuroxazide
PCNet	Network Proximity	IntAct (P-P), ChEMBL (D-P), DisGeNET (P-D), DrugCentral (D-D)	diosmin, acetylcysteine, pyrimethamine, nifuroxazide
STRING	TrustRank	NeDRex (unapproved as well)	ouabain, niclosamide, digoxin, nifuroxazide, digitoxin
STRING	Harmonic Centrality	NeDRex (unapproved as well)	ouabain, digitoxin, niclosamide, digoxin, nifuroxazide
STRING	Degree Centrality	NeDRex (unapproved as well)	niclosamide, digoxin, nifuroxazide, digitoxin, ouabain
STRING	TrustRank	STRING (P-P), NeDRex (D-P, D-D), DisGenet (P-D) (unapproved as well)	digitoxin, niclosamide, ouabain, nifuroxazide, digoxin
STRING	Harmonic Centrality	STRING (P-P), NeDRex (D-P, D-D), DisGenet (P-D) (unapproved as well)	niclosamide, ouabain, digitoxin, nifuroxazide, digoxin
STRING	Degree Centrality	STRING (P-P), NeDRex (D-P, D-D), DisGenet (P-D) (unapproved as well)	niclosamide, ouabain, digitoxin, nifuroxazide, digoxin
STRING	Network Proximity	NeDRex (unapproved as well)	nifuroxazide
STRING	Network Proximity	STRING (P-P), NeDRex (D-P, D-D), DisGenet (P-D) (unapproved as well)	nifuroxazide
PCNet	Network Proximity	STRING (P-P), NeDRex (D-P, D-D), DisGenet (P-D) (unapproved as well)	nifuroxazide
STRING	TrustRank	IntAct (P-P), ChEMBL (D-P), DisGeNET (P-D), DrugCentral (D-D) (unapproved as well)	diosmin, umifenovir, ciglitazone, nifuroxazide, acetylcysteine, pyrimethamine, genistein, aniracetam, chlorogenic acid, salvianolic acid A
STRING	Harmonic Centrality	IntAct (P-P), ChEMBL (D-P), DisGeNET (P-D), DrugCentral (D-D) (unapproved as well)	genistein, acetylcysteine, umifenovir, pyrimethamine, salvianolic acid A, nifuroxazide, ciglitazone, aniracetam, chlorogenic acid, diosmin
STRING	Degree Centrality	IntAct (P-P), ChEMBL (D-P), DisGeNET (P-D), DrugCentral (D-D) (unapproved as well)	aniracetam, genistein, pyrimethamine, chlorogenic acid, acetylcysteine, nifuroxazide, umifenovir, salvianolic acid A, diosmin, ciglitazone
STRING	Network Proximity	IntAct (P-P), ChEMBL (D-P), DisGeNET (P-D), DrugCentral (D-D) (unapproved as well)	aniracetam, nifuroxazide, umifenovir
PCNet	Network Proximity	IntAct (P-P), ChEMBL (D-P), DisGeNET (P-D), DrugCentral (D-D) (unapproved as well)	umifenovir, aniracetam

**Table 4:** In silico ADMET parameters of repurposed drug candidates.

Parameters	Entecavir	Valganciclovir	Nifuroxazide
MW	277.3	354.4	275.2
RB	4	11	6
DM	7.4	10.6	6.9
MV	844.0	1097.6	862.2
DHB	5	6	2
AHB	8.9	11.9	4.8
PSA	140.2	182.2	128.0
logP	−1.1	−1.6	1.3
logS	−2.3	−1.0	−3.3
PCaco	22.6	3.8	62.9
PM	4	5	2
%HOA	45	2	67
VRF	0	2	0
VRT	1	1	0
PAINS	0	0	0

Abbreviations: MW—molecular weight; RB—number of rotatable bonds; DM—dipole moment; MV—total solvent-accessible volume; DHB—estimated number of hydrogen-bond donors; AHB—estimated number of hydrogen-bond acceptors; PSA—van der Waals polar surface area; logP—predicted octanol/water partition coefficient; logS—predicted aqueous solubility; PCaco—predicted Caco-2 cell permeability (nm/s); PM—number of predicted metabolic reactions; %HOA—predicted percentage of human oral absorption; VRF—Lipinski’s rule of five (violation count); VRT—Jorgensen’s rule of three (violation count); PAINS—pan-assay interference compound alerts.

## Data Availability

The data that support the findings of this study are available from the corresponding authors upon reasonable request.

## Supplementary Materials

The following supporting information can be downloaded at https://www.mdpi.com/article/10.3390/pharmaceutics17081021/s1. Figure S1. Network-based visualization of drug repurposing candidates for kala-azar generated using the Drugst.One platform. Figure S2. Top-ranked approved drugs identified by network pharmacology as repurposing candidates for kala-azar. Figure S3. Graphical representation of the associations between disease-relevant genes and drug candidates identified for kala-azar.
