# Supplementary material for: Drug Repurposing for Kala-Azar
Source: Pharmaceutics. 2025 Aug 6;17(8):1021. doi: 10.3390/pharmaceutics17081021 (PMC12388976; doi:10.3390/pharmaceutics17081021)
Supplement: Supplementary file 1 [file pharmaceutics-17-01021-s001.zip › pharmaceutics-3747330-supplementary.pdf]

Supplementary Materials

Drug repurposing for kala-azar

Biljana Arsić[1,2,*], Budimir S. Ilić[3,*], Andreas Maier[2], Michael Hartung[2], Jovana Janjić[4], Jelena Milićević[5], Jan Baumbach[2,6]

[1] University of Niš, Faculty of Sciences and Mathematics, Department of Chemistry, Višegradska 33, Niš, Republic of Serbia

[2] University of Hamburg, Institute for Computational Systems Biology, Albert-Einstein-Ring 8-10, Hamburg, Germany; andreas.maier-1@uni-hamburg.de (A.M.); michael.hartung@uni-hamburg.de (M.H.); jan.baumbach@uni-hamburg.de (J.B.)

[3] University of Niš, Faculty of Medicine, Department of Chemistry, Blvd. Dr Zorana Đinđića 81, Niš, Republic of Serbia

[4] University of Belgrade, Faculty of Biology, Studentski trg 16, Belgrade, Republic of Serbia; jovanajanjicc@gmail.com (J.J.)

[5] University of Belgrade, Institute of Nuclear Sciences "Vinča", Laboratory for Bioinformatics and Computational Chemistry, Mike Petrovića Alasa 12-14, Belgrade, Republic of Serbia; jdjordjevic@vin.bg.ac.rs (J.M.)

[6] Computational Biomedicine Lab, Department of Mathematics and Computer Science, University of Southern Denmark, Odense, Denmark

* Correspondence: biljana.arsic@pmf.edu.rs (B.A.) and budimir.ilic@medfak.ni.ac.rs (B.S.I.)

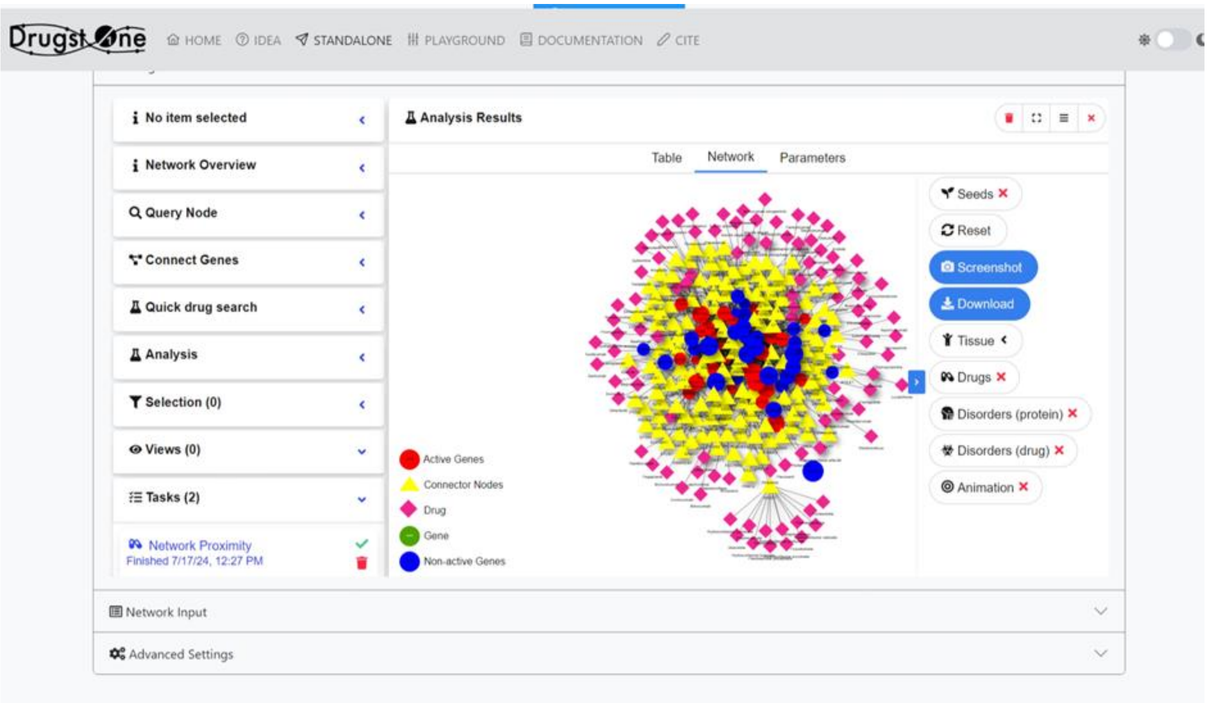

Figure S1. Network-based visualization of drug repurposing candidates for kala-azar generated using the Drugst.One platform.

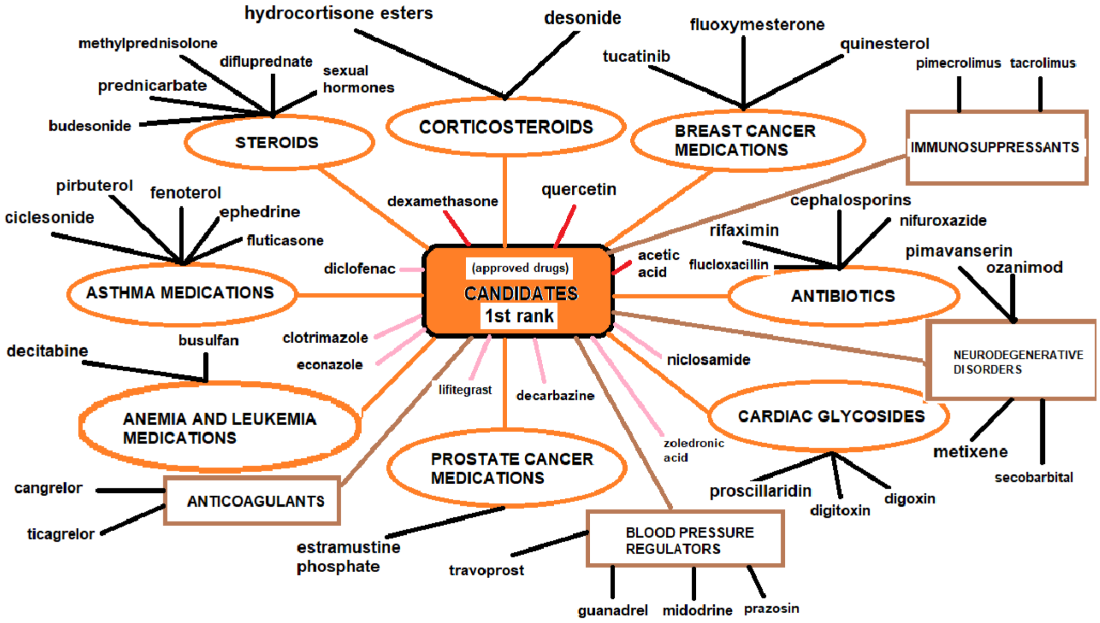

Figure S2. Top-ranked approved drugs identified by network pharmacology as repurposing candidates for kala-azar.

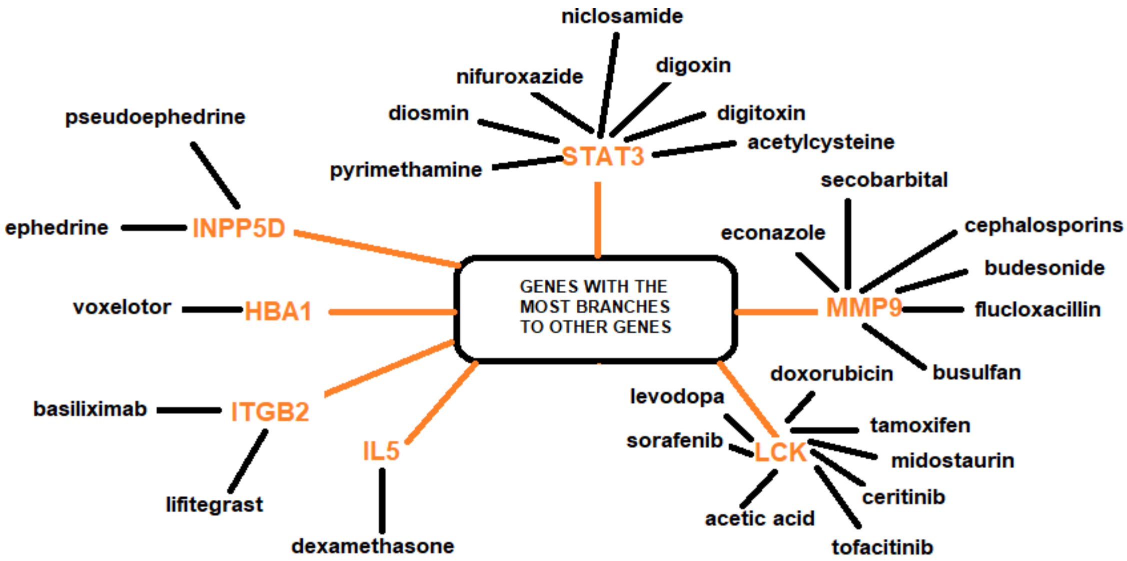

Figure S3. Graphical representation of the associations between disease-relevant genes and drug candidates identified for kala-azar.
